# Development of a prognostic signature based on immune-related genes and the correlation with immune microenvironment in breast cancer

**DOI:** 10.18632/aging.204158

**Published:** 2022-07-05

**Authors:** Menglu Dong, Xiaoqing Cui, Ge Wang, Qi Zhang, Xingrui Li

**Affiliations:** 1 Department of Thyroid and Breast Surgery, Tongji Hospital, Tongji Medical College, Huazhong University of Science and Technology, Wuhan 430030, China; 2 Department of Plastic and Cosmetic Surgery, Tongji Hospital, Tongji Medical College, Huazhong University of Science and Technology, Wuhan 430030, China

**Keywords:** breast cancer, immune-related gene, prognosis, risk model, immune infiltration

## Abstract

Breast cancer (BC) is an inflammatory tumor caused by a variety of pathological factors, and is still the most common malignant tumor in women. Immune-related genes (IRGs) play a prominent role in the oncogenesis and progression of BC, and are of tumor-specific expression patterns that would benefit the prognosis evaluation. However, there were no systematic studies concerning the possibilities of IRGs in BC prognosis. In this study, the Cancer Genome Atlas (TCGA) database was used to integrate the expression profiles of IRG with the overall survival (OS) rate of 1039 breast cancer patients. The Cox regression analysis was used to predict the survival-related IRGs in BC. Then, we successfully screened a total of 6 IRGs, including PSME2, ULBP2, IGHE, SCG2, SDC1, and SSTR1, and accordingly constructed a prognosis prediction model of BC. Based on the IRG-related model, the BC patients were divided into high- and low-risk groups, and the association between the prognostic model and tumor immune microenvironment (TME) was further explored. The prognostic model reflected the infiltration of various immune cells. Moreover, the low-risk group was found to be with higher immunophenoscore and distinct mutation signatures compared with the high-risk group. The histological validation showed that SDC1, as well as M2 macrophage biomarker CD206, were both of higher abundance in BC samples of high-risk patients, compared with those of low-risk patients. Our results identify the clinically significant IRGs and demonstrate the importance of the IRG-based immune prognostic model in BC monitoring, prognosis prediction, and therapy.

## INTRODUCTION

Nowadays, breast cancer (BC) has transcended lung cancer as the most common female cancer worldwide, accompanied by approximately 2.3 million new cases (11.7%) [1]. It is worth noting that BC metastasis, which is a complex, multistage process and is prone to colonize to the distant brain, lung, and bone, accounting for the leading cause of death from BC [2]. Currently metastasis and treatment resistance are the main challenge in BC therapy and are intensively associated with cancer relapse post-treatment [3]. Early and rapid detection of BC with accurate and efficient diagnosis is very irreplaceable in clinical practice. Therefore, there remains an urgent need to explore and investigate new diagnostic and risk models, which are essential for individualized treatment and prognostic prediction of BC.

The systematically developed prognostic models for diseases have received numerous attention, particularly in cancer prognosis. Generally, these models are composed of signatures and patterns by including tumor-specific mRNAs, non-coding RNAs, and proteins [4]. Notably, immune-related genes (IRGs) play a multifaceted role in the promoting or suppressing BC oncogenesis and progression [5]. The IRGs, typically represented by programmed death 1/programmed cell death-ligand 1 (PD1/PD-L1), have attracted much attention in tumor immunotherapy in BC [6]. In addition, the progression of BC has been implied to be closely related to the tumor immunophenotype [7]. Hence, it is not a stretch to infer that a robust, reliable, and individualized IRG-based classifier can be highly valuable for predicting BC outcomes.

For judging the prognosis of colorectal cancer (CRC), Wen et al. reported a superior risk model comprised of eight IRGs (SLC10A2, UTS2, FGF2, UCN, IL1RL2, ESM1, ADIPOQ, and VIP) [8]. Their result showed that the overall survival (OS) in the high-risk group was markedly lower than that in the low-risk group. It emphasized the excellent capability of IRGs in predicting the clinical outcomes in CRC. Ren et al. also constructed a prognostic model based on six IRGs, which monitor and predict the prognosis of clear cell renal cell carcinoma [9]. Meanwhile, this model was significantly related to the clinicopathological feature, as well as various immune cell infiltration in the tumor microenvironment (TME). Additionally, an interesting study of the prognostic model based on 5 IRGs, including ERAP2, CXCL9, AREG, DKK1, and IL20RB, deciphered that the high-risk patients according to risk score had a poorer survival and a significantly higher characteristic immune checkpoint profile, in comparison to the low-risk patients in the setting of pancreatic cancer [10]. Moreover, this model could reflect the infiltration abundance of neutrophils and dendritic cells (DCs). Thus, all these results directly proved that IRG-based models provide valuable information for the survival prediction in various cancer types. More importantly, IRG-related risk models identify an association between genes and characteristics of immune infiltration, conferring the potential for immunotherapy response and personalized treatment in BC patients.

Nevertheless, although some studies have utilized the algorithms to predict prognostic or IRG-related survival in BC, there were no systematical studies on the possibilities of IRGs in BC prognosis. Hence, to address this issue, we intended to screen and validate molecular markers that could effectively predict survival in BC patients. Firstly, we screened out a total of 6 IRGs and accordingly established a prognostic risk scoring model. This risk model could successfully classify the BC patients into high- and low-risk groups. Then, we evaluated the risk score in assessing the correlation between candidate IRGs and prognostic value, clinicopathological characteristics, functional enrichment analysis, and tumor-infiltrating immune state. Finally, with comprehensive genomic database analysis, this robust immune-related prognosis model possessed excellent predictive ability in BC prognosis and characterization of immune infiltration. Together, this well-established risk model could precisely predict the prognosis of BC patients, posing a pivotal biomarker-encompassing pattern for the immune therapy and prognostic evaluation of patients with BC.

## MATERIALS AND METHODS

### Data acquisition and preprocessing

**Figure 1:**
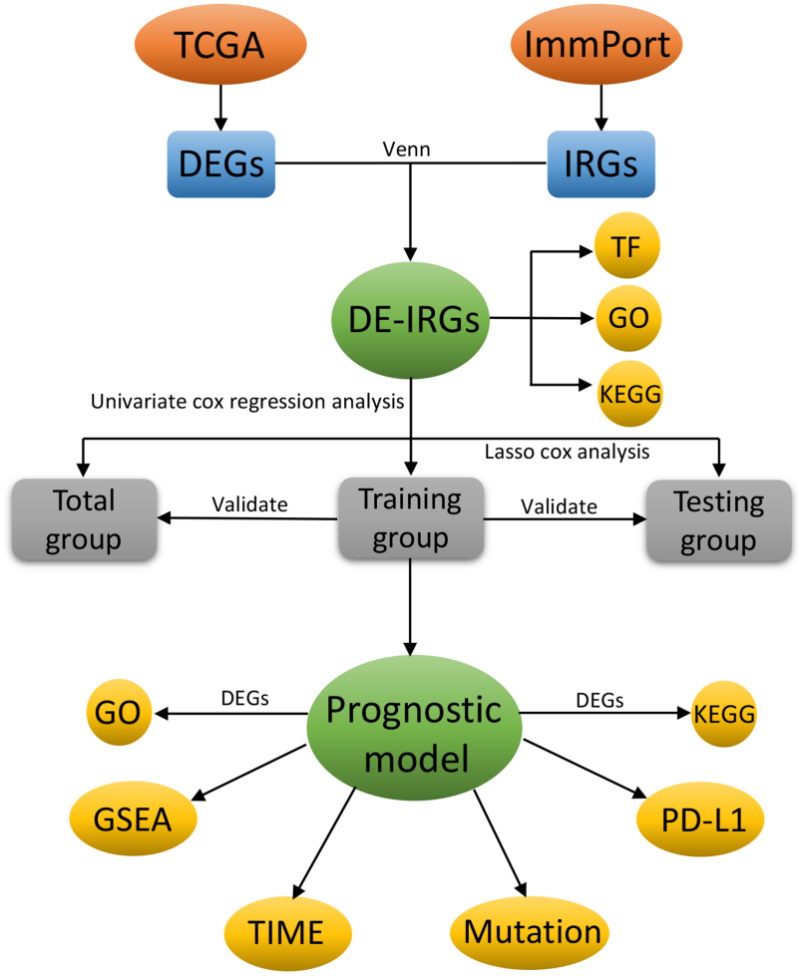
The flowchart of this study.

The RNA-sequencing datasets and the corresponding clinical characteristics data of BC patients were downloaded from The Cancer Genome Atlas (TCGA) database (https://cancergenome.nih.gov/). Patients with less than 30 days of follow-up and male BC patients were excluded. Finally, 1039 patients were included in this study and randomized into training group (*n* = 520) and testing group (*n* = 519) by using the R package “caret” for subsequent analysis [11]. The training set was used to construct the prognostic immune gene signature, while the entire set and testing set were used to validate the predictive ability of the established prognostic immune model. The list of IRGs was downloaded from the Immunology Database and the Analysis Portal (ImmPort) database (https://www.immport.org/) [12], an open platform of human immunology database for clinical and translational research. These genes could be identified and extensively participate in the important process of immunology, thus providing a good foundation for immunology research (Figure 1).

### Differentially expressed genes (DEGs) and IRGs analysis

Then, we performed the DEG analysis on the transcriptional data from the TCGA database with cutoff values of false discovery rate (FDR) < 0.05 and log2 |fold change| > 1. After that, we extracted the differentially expressed IRGs from the above DEGs and used the limma package of R software (http://bioconductor.org/packages/limma/) to acquire the differentially expressed IRGs associated with BC between cancer and adjacent non-tumor samples obtained differentially expressed IRGs associated with BC using the limma package of R software (http://bioconductor.org/packages/limma/). Finally, the Kyoto Encyclopedia of Genes and Genomes (KEGG) enrichment pathway analysis and the Gene Ontology (GO) annotation were utilized to explore the underlying molecular mechanism of these differentially expressed IRGs.

### Extract Transcription factors (TFs) and construct the regulatory network

The Cistrome Cancer Database summarizes the TCGA genome data accompanied with more than 23,000 chip-SeQ and chromatin accessibility maps to demonstrate regulatory relationships between TFs and genes [13]. The OS time of patients was obtained by downloading clinical information from the TCGA database. The univariate Cox analysis was performed by using the R survival package to screen out the IRGs that were closely related to survival prognosis. Accordingly, regulatory network was constructed for seeking the interrelation between these genes and TFs. In this study, TFs were compared with previously obtained IRGs to screen differentially expressed TFs related to survival-related IRGs. The regulatory network diagram of TFs and survival-related IRGs was drawn using Cytoscape software (version 3.8.0) [14].

### Construction and verification of the immune-related prognostic signature

To construct an IRG-based immune-related prognostic model, genes significantly associated with prognosis were screened by univariate Cox regression analysis, and risk coefficients were obtained by Lasso-Penalized Cox regression analysis. For predicting OS, BC patients were divided into a high-risk group and low-risk group based on the calculation results of the R packages “survival” and “survminer”, using the median risk score as the best cut-off value. The time-dependent prognostic capability of the gene signature was measured by calculating the area under the curve (AUC) [15], using the R package “survivalROC” [16]. In addition, univariate and multivariate Cox regression analyses were performed to evaluate the prognostic significance and routine clinicopathological features, including age, grade, clinical stage, and TNM stage. These results were presented through the R package “Ggpubr” [17]. The principal components analysis (PCA) is used as a statistical method to find key variables in multidimensional datasets. PCA could predict the analysis and visualize of multidimensional data sets [18] through limma [19] and scatterplot 3d [20] packages. Subsequently, PCA was performed to verify the grouping ability of signatures by identifying a small set of synthetic variables through a dimensionality reduction process. Finally, the m6Ascore was determined in a manner similar to the Genomic Grade Index (GGI) [21]: *riskScore* = ∑ (*PC*1_*i*_ + *PC*2_*i*_), where *i* was the expression of overlapping IRGs with a significant difference in prognosis of BC patients.

### Gene set enrichment analysis (GSEA)

GSEA is a computational method for identifying whether defined gene sets are statistically significantly and consistently different across biological states [22]. We performed GESA using the JAVA program (https://www.broadinstitute.org/gsea), to explore the potential KEGG pathway enrichment terms of the IRG signature in the BC cohort. A total of 1000 random sample permutations were included, and enriched gene sets with nominal *p* < 0.05 and FDR < 0.25 were considered statistically significant.

### Comparison of TME cell infiltration among high- and low-risk group

To understand the degree of immune cell infiltration in the two subgroups, the relative abundance of each cell infiltration in the TME of BC samples was quantified using single-sample gene-set enrichment analysis (ssGSEA). From the research of Charoentong, we obtained the gene sets for each type of TME infiltrating immune cells, and stored a relatively comprehensive subset of human immune cells, including activated CD8 T cells, natural killer T cells, activated DCs, macrophages, and regulatory T cells.

### BC sample validation experiments

Here, we adopted the immunofluorescence (IF) and immunohistochemistry (IHC) to verify one of the model-associated IRGs (SDC1) in the collected BC samples. The BC tumor samples were Surgically excised in our department of thyroid and breast surgery (Tongji Hospital). Specifically, for immunohistochemistry (IHC), all the BC tissues were deparaffinized and were heated in citrate buffer in sequential. Then, the obtained sections were immune-stained with a primary anti-SDC1 antibody (CD138/Syndecan-1 Rabbit pAb, ABclonal, China Catalog: A1235) overnight at 4°C. After washing, these incubated sections were then incubated with horseradish peroxidase (HRP)-conjugated secondary antibodies. DAB peroxidase substrate Kit (Maxin, China) was used to observe peroxidase activity and sections were restained with hematoxylin. Digital images of sections were collected by SOPTOP CX40 microscope (China). The IF experiment was conducted to verify the distribution of SDC1 and its correlation with M2 macrophages in the BC samples. For IF, the obtained sections were incubated with the above anti-SDC1 antibody, anti-CD2062 antibody, and nuclear 4,6-diamidino-2-phenylindole (DAPI, Sigma, USA) for counterstaining. Digital image acquisition was realized by using a fluorescence microscope (Olympus, Japan).

### Statistical analysis

All the statistical analyses were performed by the R (v.3.6.3) software. The Fisher’s exact test or Pearson χ^2^ test was used to analyze qualitative variables as appropriate. *P* < 0.05 was considered statistically significant.

### Availability of data and materials

The datasets used and/or analyzed during the present study are available from the corresponding author on reasonable request.

## RESULTS

### Data sources and identification of differentially expressed IRGs

**Figure 2:**
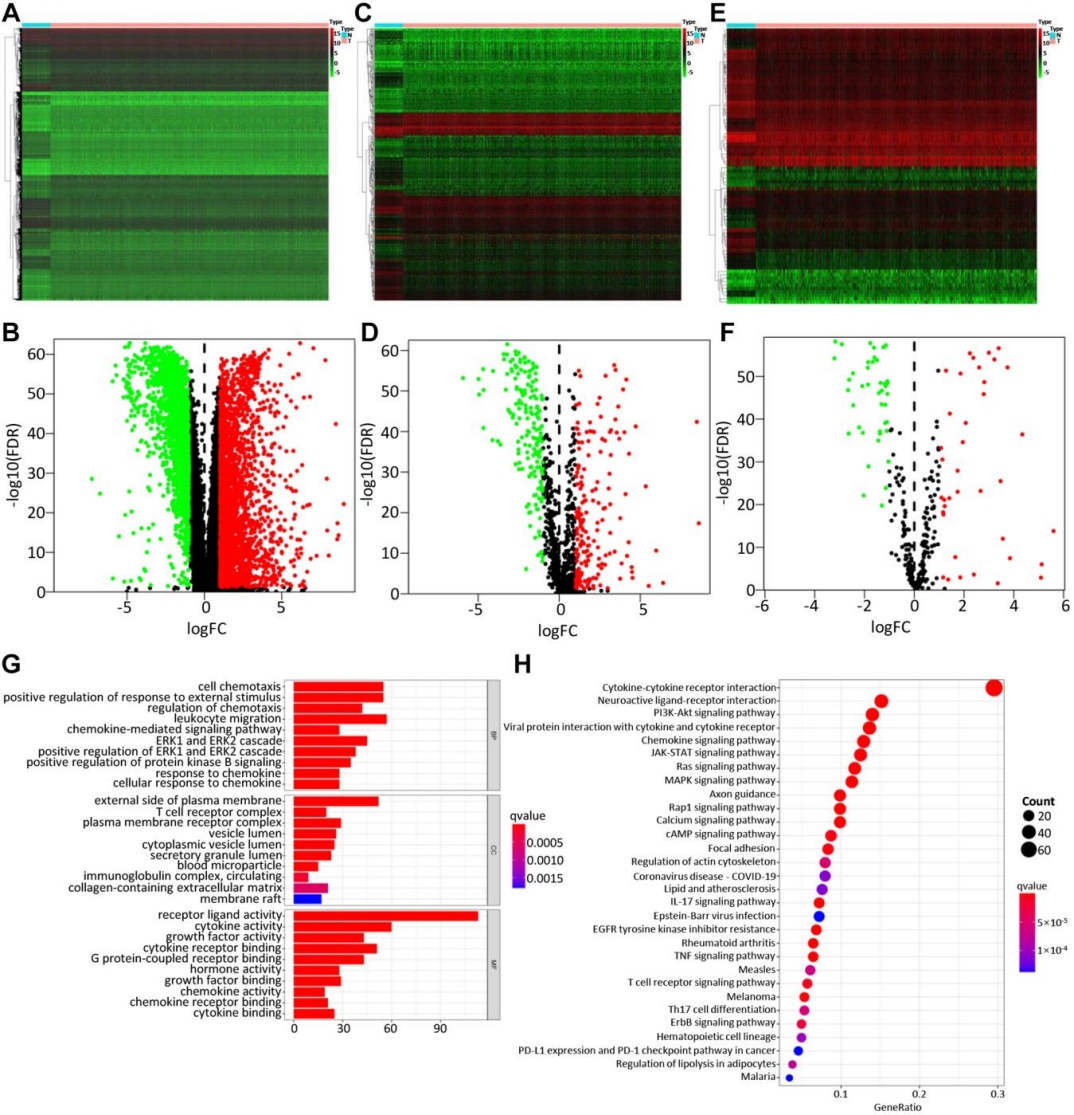
**Differentially expressed genes, immune-related genes, and TFs.** The differentially expressed genes between BC and non-tumor tissues were shown in the heatmap (**A**) and the volcano plot (**B**). Heatmap (**C**) and the volcano plot (**D**) indicated the differentially expressed immune-related genes (IRGs). Heatmap (**E**) and volcano plot (**F**) referred to the differential TFs between breast cancer and non-tumor tissues. Red dots represented the upregulated genes or TFs, green dots represent downregulated genes or TFs, and black dots represented not differentially expressed genes or TFs. N, normal tissue. T, tumor. The GO (**G**) and KEGG (**H**) functional enrichment analysis of immune-related genes (IRGs).

A total of 4575 DEGs were identified from 1109 BC samples and 113 normal samples, including 2698 up-regulated and 1877 down-regulated genes (Figure 2A, 2B). Among them, 366 differentially expressed IRGs (193 up-regulated and 173 down-regulated) were obtained from these DEGs by immune gene co-expression analysis using the ImmPort database (Figure 2C, 2D). The result of GO analysis showed that the differentially expressed IRGs were enriched in several biological processes, including cellular response to chemokine, leukocyte migration, and chemokine-mediated signaling pathway. Cellular component analysis demonstrated that IRGs were mostly enriched in T cell receptor complex, immunoglobulin complex, and circulating, cytoplasmic vesicle lumen. And in the molecular function, these genes were mostly enriched in receptor-ligand activity and cytokine activity (Figure 2G). The KEGG analysis indicated that IRGs were mainly involved in Cytokine-cytokine receptor interaction (Figure 2H).

### Identification of survival-related IRGs and construction of the TF regulatory network

By integrating the mRNA expression and clinical information of BC patients in the TCGA database, we finally screened 39 IRGs with significant differences in the prognosis of BC patients (*P* < 0.05) (Supplementary Table 1). Then, to further explore the potential molecular mechanism of these survival-related IRGs (Supplementary Figure 1A), the expression patterns of 318 TFs were examined and 80 TFs were found to be differentially expressed in BC and non-tumor breast tissues in the TCGA database (Figure 2E, 2F). Subsequently, we analyzed the relationship between prognostic immune genes and differentially expressed TFs, and screened out 22 TFs and 23 prognostic immune genes, thus constructing a regulatory network with a correlation score > 0.4 and *p*-value < 0.001. In particular, the visualization of TF-based regulatory network clearly showed the interactions between these genes (Supplementary Figure 1B).

### Construction of the immune-related prognostic model

**Figure 3:**
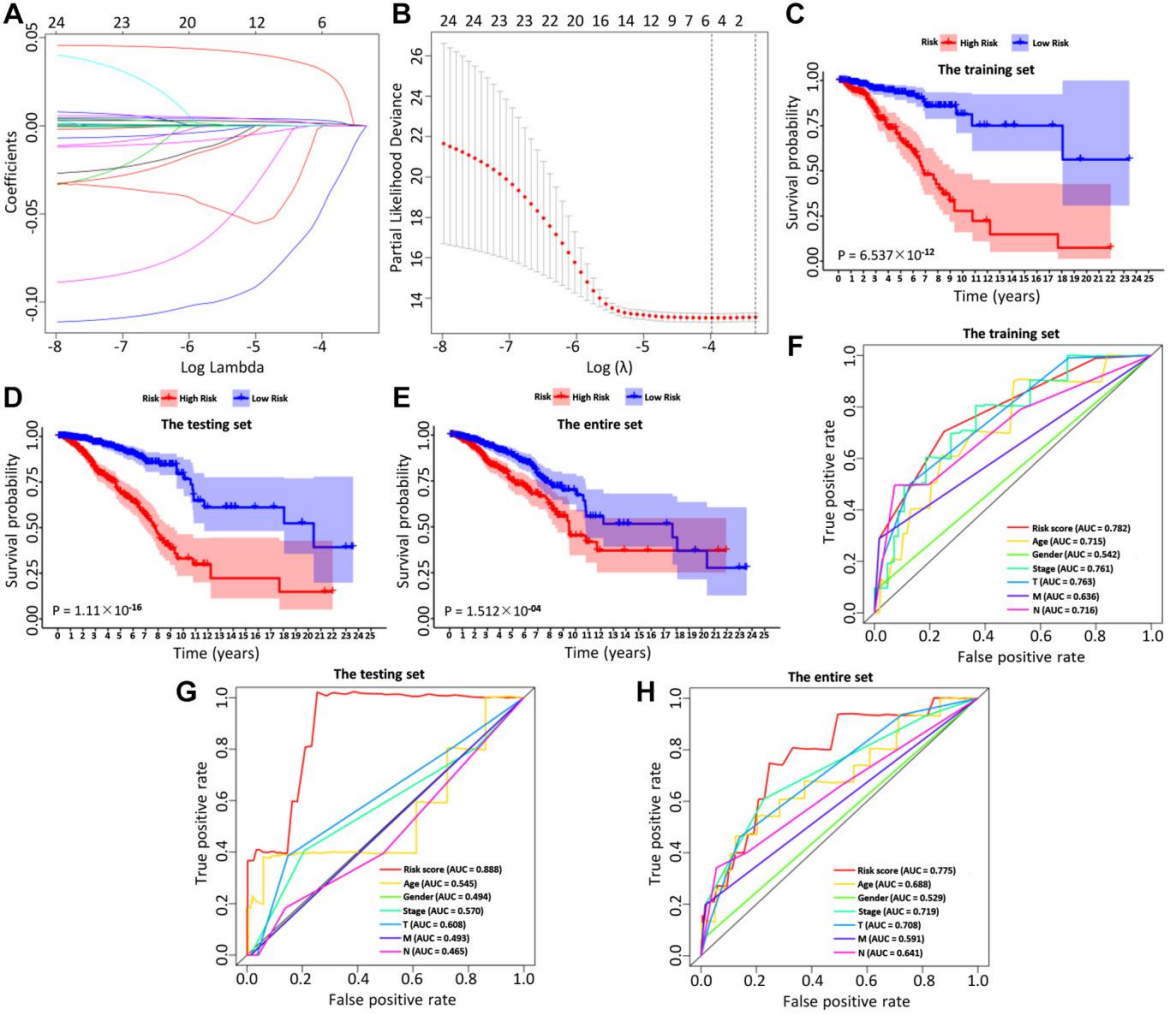
The LASSO coefficient profiles about 6 immune-related genes were shown in (**A**, **B**). The lower X-axis indicated log (λ), the upper X-axis indicated the average number of OS-related genes, and the Y-axis showed the partial likelihood deviance error. Red dots indicated the average partial likelihood deviances about the model with a given λ, the vertical bars represented the range of the partial likelihood deviance errors, and the vertical black dotted lines meant the best fit with the optimal λ values. The training set (**C**), the testing set (**D**) and the entire set (**E**) showed the survival curves of high-risk group and low-risk group respectively. The survival-dependent receiver operating characteristic (ROC) curves prognostic value in 1 year of the three sets were shown in the (**F**–**H**).

BC patients were randomly divided into a training set and a test set, including 520 and 519 BC patients, respectively. By performing univariate Cox regression on the training data, it found that 40 IRGs were retained in the training data with *p* < 0.05 (Table 1, Supplementary Table 2). The Cox regression analysis and least absolute shrinkage selection operator (LASSO) regression were used to analyze the expression profiles of differentially expressed IRGs in the training group, to define the candidate genes (Figure 3A, 3B). Finally, a total of 6 IRGs were obtained, including PSME2, ULBP2, IGHE, SCG2, SDC1, and SSTR1. The specific calculation formula was as follows: [Expression level of PSME2^*^ (−0.0143)] + [Expression level of ULBP2^*^ (0.1070)] + [Expression level of IGHE^*^ (0.0619)] + [Expression level of SCG2^*^ (0.0044)] + [Expression level of SDC1^*^ (0.0021)] + [Expression level of SSTR1^*^ (0.0451) (Table 2). Based on these prognostic genes, we successfully established the immune-related prognosis model for OS.

**Table 1:** Univariate Cox proportional hazards regression analysis of the top 20 IRGs.

**ID**	**HR**	**HR.95L**	**HR.95H**	***p*-value**
**IGHE**	1.059742	1.031821	1.088418	2.05E-05
**SCG2**	1.004126	1.002015	1.006241	0.000125
**SSTR1**	1.042095	1.020016	1.064653	0.000161
**ULBP2**	1.124144	1.053613	1.199397	0.000401
**PSME2**	0.985116	0.97654	0.993767	0.000775
**SDC1**	1.002568	1.001053	1.004085	0.000888
**CCL24**	1.090964	1.031501	1.153854	0.00233
**MMP9**	1.000283	1.0001	1.000467	0.002478
**TRDV1**	0.665198	0.496953	0.890401	0.006139
**FLT3**	0.88369	0.808757	0.965565	0.006237
**TNFRSF8**	0.512282	0.311931	0.841316	0.008227
**PLAU**	1.005204	1.001327	1.009097	0.008479
**IL18**	0.935109	0.888893	0.983728	0.009476
**TSLP**	0.192396	0.054878	0.674515	0.010019
**TNFSF4**	1.121123	1.026726	1.224199	0.010844
**NPR3**	1.036536	1.007298	1.066621	0.013968
**ADM**	1.017638	1.0033	1.032182	0.015736
**TRBC2**	0.982984	0.96928	0.996882	0.016577
**CXCL9**	0.996502	0.993567	0.999445	0.019878
**TRBV28**	0.968825	0.943218	0.995128	0.020489

**Table 2:** Six immune-related genes identified from TCGA by Cox regression analysis.

**ID**	**coef**	**HR**	**HR.95L**	**HR.95H**	***P*-value**
**PSME2**	−0.01427717	0.98582426	0.97744488	0.99427548	0.001045
**ULBP2**	0.106960761	1.11289059	1.03636684	1.19506473	0.00325327
**IGHE**	0.061897277	1.06385306	1.03658715	1.09183615	2.97E-06
**SCG2**	0.004393524	1.00440319	1.00225846	1.00655251	5.62E-05
**SDC1**	0.002059167	1.00206129	1.00045621	1.00366894	0.01181509
**SSTR1**	0.045059224	1.04608981	1.02363949	1.06903252	4.69E-05

### Validation of the immune-related prognostic model

**Figure 4:**
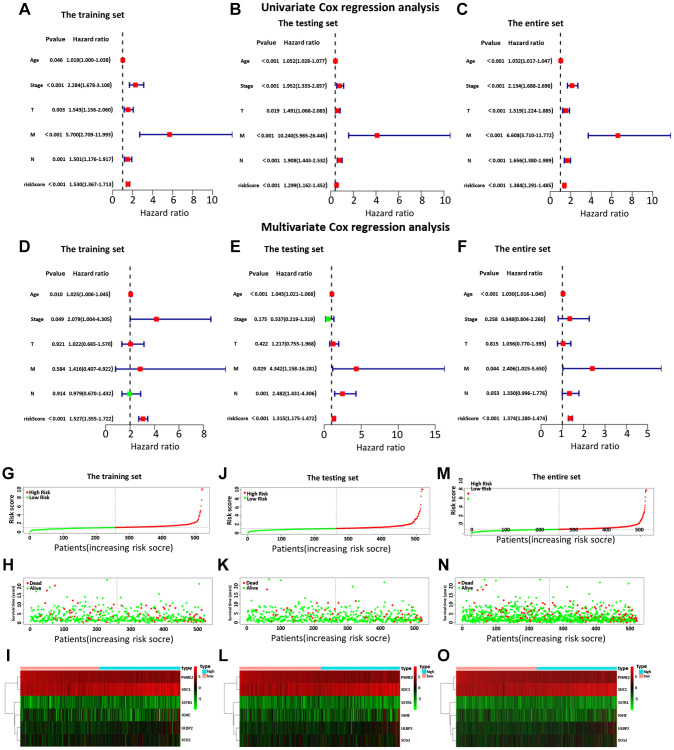
The univariate (**A**–**C**) and multiple (**D**–**F**) regression analysis of BC, including the relationships of the age, stage, T stage, distant metastasis, lymph node metastasis, and riskScore in the three sets. The training set (**A**, **D**), the testing set (**B**, **E**), and the entire set (**C**, **F**). The green squares indicated that the median value of hazard ratio (HR) was less than 1, while the red squares indicated that the median value of HR was greater than 1. Analysis of risk score, OS, and the expression of the six genes in the training set (**G**–**I**), testing set (**J**–**L**), and entire set (**M**–**O**). The risk score, OS, and heat map were listed from top to bottom.

Then, we examined the comparisons of survival differences between the high- and low-risk group in the training set (Figure 3C), the testing set (Figure 3D), and the entire set (Figure 3E). The Kaplan-Meier log-rank analysis revealed significant differences in OS between the two risk groups. In addition, we found that the AUCs of OS in training set, test set and entire set were 0.782, 0.888, and 0.775, respectively (Figure 3F–3H). Our IRG model had the maximum AUC value compared to other clinical features, demonstrating good predictive power. Subsequent risk curve, scatter diagram, and heatmap were used to analyze the risk score distribution (Figure 4G–4I), survival status (Figure 4J–4L), and the expression of the candidate genes (Figure 4M–4O) for each BC patient in the training set, testing set, and the entire set, respectively. The univariate and multivariate Cox regression analysis verified that the IRGs in the prognosis model could serve as independent predictors of prognosis (Figure 4A–4F).

**Figure 5:**
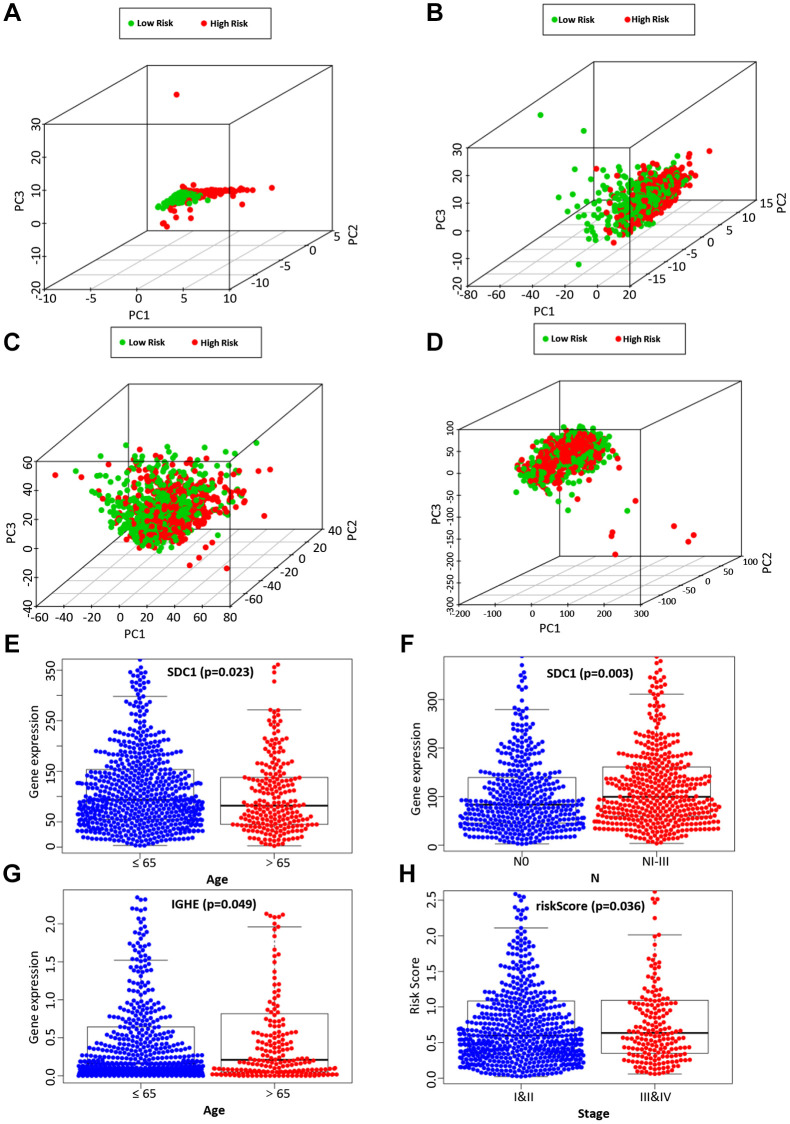
The PCA based on the immune-related signature (**A**), immune-related genes (**B**), differently expressed genes (**C**), and the entire gene expression profiles (**D**) between the high-risk group and low-risk group. The correlation of the immune-related signature with clinicopathological characteristics. SDC1 was associated with age (**E**) and lymph node metastasis (**F**). IGHE were associated with age (**G**). The risk score of our prognostic model was significantly associated with a higher tumor stage (**H**).

Next, by analyzing the model and different clinicopathological factors, the prognosis of low-risk group was significantly better than that of high-risk group in terms of age (≤ 65/ > 65) (Supplementary Figure 2A and 2B), T stage (T1 + T2/T3 + T4) (Supplementary Figure 2C and 2D), and stage (I + II/III + IV) (Supplementary Figure 2E and 2F). Similarly, the patients without lymph node metastasis (Supplementary Figure 2G) and distant metastasis (Supplementary Figure 2H and 2I) had a similar outcome. The PCA was further to detect the difference between the low-risk group and the high-risk group according to the immune-related signals (Figure 5A), immune genes (Figure 5B), differential expression genes (Figure 5C), and the entire gene expression profiles (Figure 5D). As seen in Figure 5, compared with the other three groups, the high-risk group and the low-risk group in Supplementary Figure 2A were distributed in different directions, proving that our prognosis model could effectively distinguish the high-risk group from the low-risk group.

### Assessment of the correlation between candidate genes and clinicopathological characteristics

We then analyzed the relationship between the expression of 6 candidate IRGs and different clinical features, including age, gender, tumor grade, T stage, clinical stage, lymph node metastasis, and distant metastasis. Among 6 candidate IRGs, IGHE and SDC1 were correlated with age, but IGHE expression was increased in patients over 65 years old, while SDC1 expression was increased in patients under 65 years old (Figure 5E, 5G). Besides, the expression difference of SDC1 between lymph node metastasis groups was statistically significant (Figure 5F). Finally, the risk score based on our prognostic model was significantly associated with the higher tumor grade (Figure 5H).

### Different states of functional enrichment analysis between high-risk and low-risk groups

**Figure 6:**
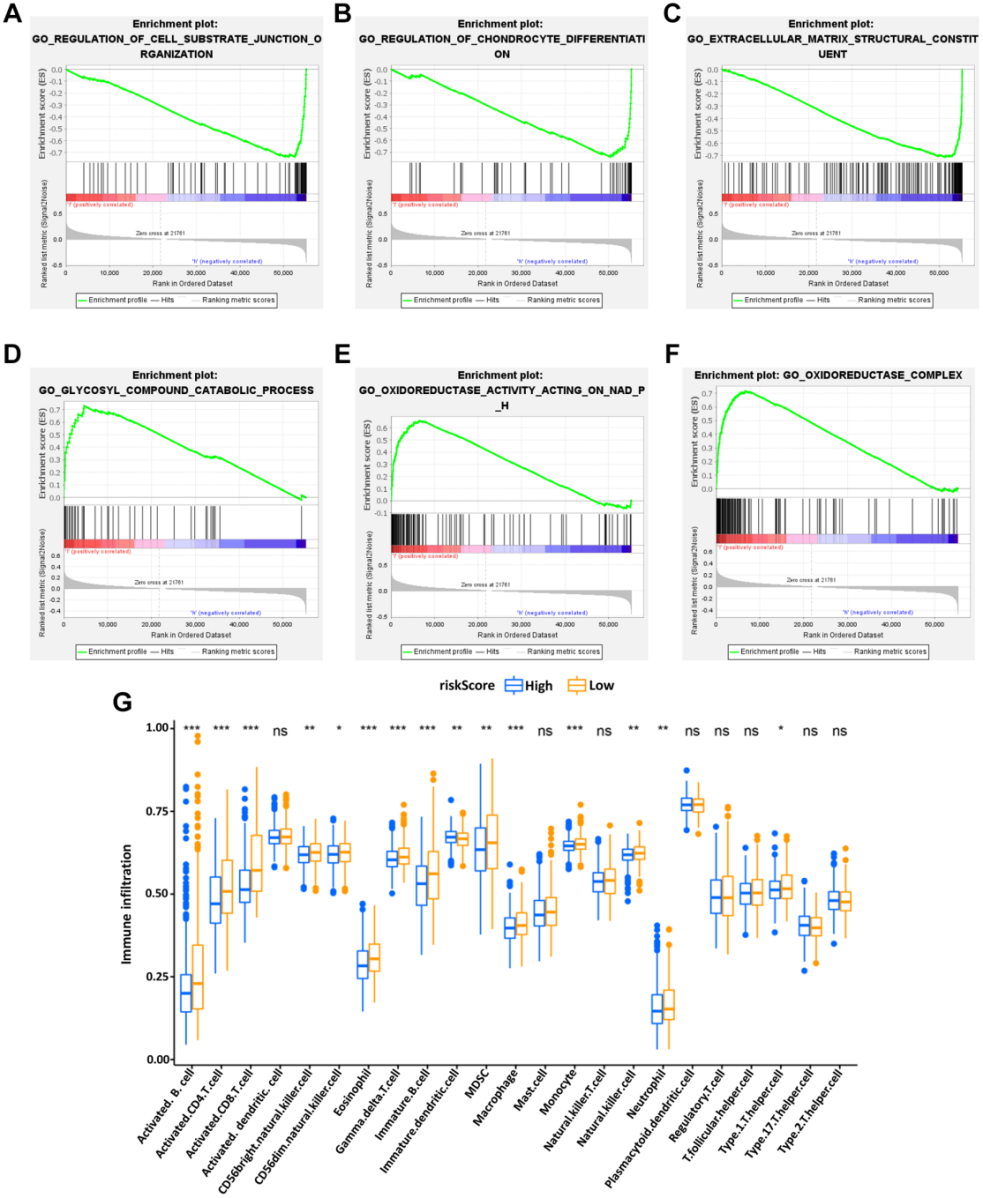
**Enrichment plots of Gene Ontology annotation from gene set enrichment analysis (GSEA).** GSEA results showed that the regulation of cell substrate junction organization (**A**), Regulation of chondrocyte differentiation (**B**), and Extracellular matrix structural constituent (**C**) were differentially enriched in low-risk phenotype, while Glycosyl compound catabolic process (**D**), Oxidoreductase activity on NADPH (**E**) and Oxidoreductase complex (**F**) were enriched in the high-risk phenotype. The expression abundance of different TME infiltrating cells in the high- and low-risk group (**G**). The upper and lower end of the box represented the quartile range of the value, the middle line represented the median value, and the asterisk represented the statistical *p*-value (^*^P < 0.05, ^**^P < 0.01, ^***^P < 0.001).

GSEA was used to investigate the differences between the high- and low-risk groups. The results revealed that the GO biological process “Regulation of cell substrate junction organization” (Figure 6A) and “Regulation of chondrocyte differentiation” (Figure 6B), molecular function “Extracellular matrix structural constituent” (Figure 6C) were differentially enriched in low-risk groups (*P* < 0.01). Besides, the biological process “Glycosyl compound catabolic process” (Figure 6D), molecular function “Oxidoreductase activity acting on NADPH” (Figure 6E) and cellular component “Oxidoreductase complex” (Figure 6F) were associated with the high-risk groups (*P* < 0.01).

Besides, the KEGG pathway analysis showed that the genes of low-risk group were mainly enriched in the “TGF beta signaling pathway” (Supplementary Figure 3A), “Hedgehog signaling pathway” (Supplementary Figure 3B), and “Adherens junction” (Supplementary Figure 3C) (*P* < 0.01) while the “Antigen processing and presentation” (Supplementary Figure 3D), “Cytosolic DNA sensing pathway” (Supplementary Figure 3E) and “Oxidative phosphorylation” (Supplementary Figure 3F) were enriched in high-risk group (*P* < 0.01). The function enrichment of IRGs between high- and low-risk groups was explored, showing that the IRGs in our model were mainly involved in immune-related signaling pathways (Supplementary Figure 1C, 1D).

### The tumor-infiltrating immune cells in risk signature

Subsequently, to explore the relationship between the IRG-based prognostic risk model and TME, the differences in tumor-infiltrating immune cells between the high- and low-risk groups defined in our prognostic model were analyzed. It was found that the abundance of activated B cells, activated CD4 T cells, immature DCs and eosinophils were significantly more abundant in the low-risk group of the OS model (Figure 6G). These results potentially shed light on the regulatory mechanisms of BC TME.

### The immune-related risk signature and mutation profile

**Figure 7:**
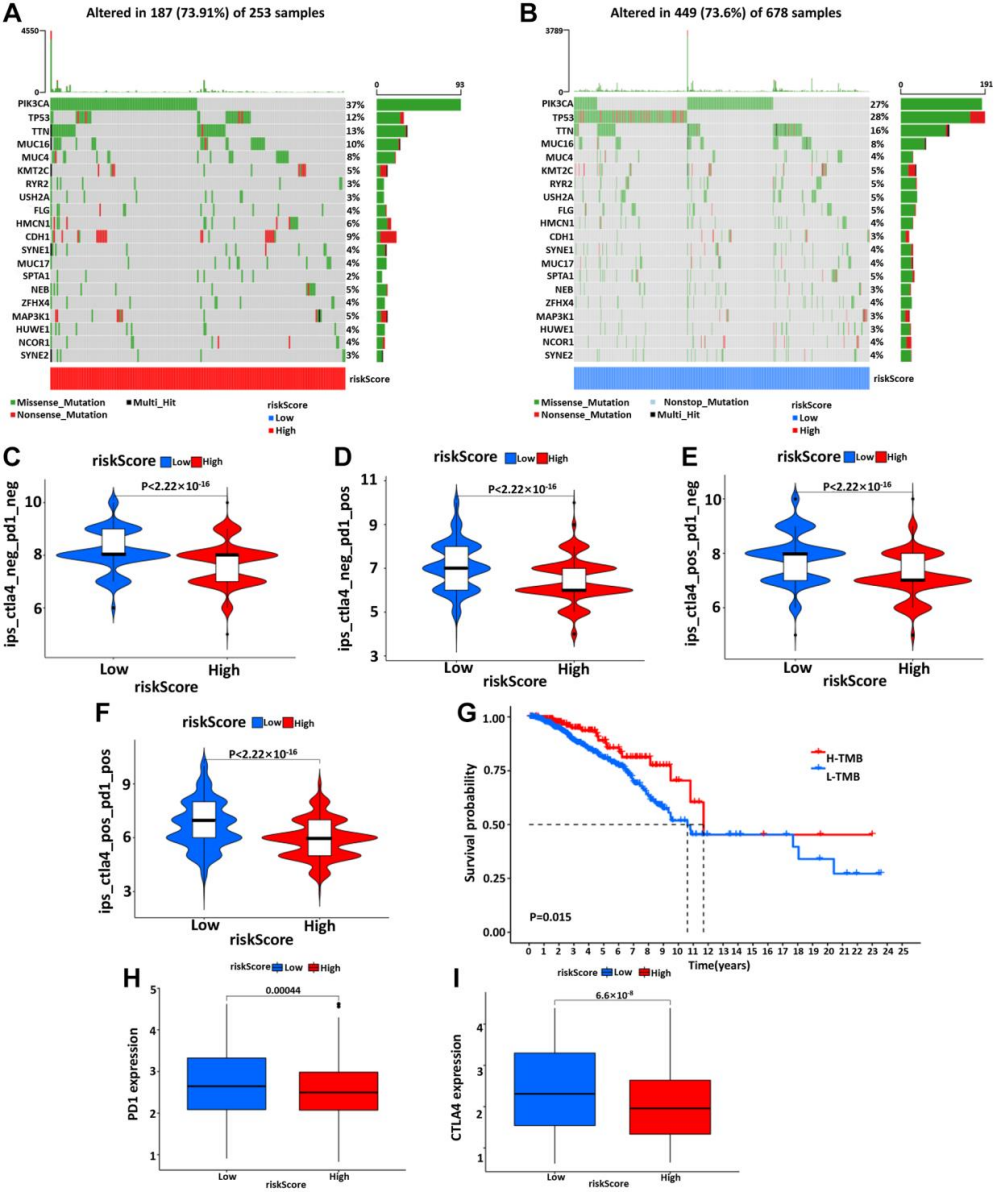
The waterfall diagram of tumor somatic mutation in patients with high- (**A**) and low- (**B**) risk groups. (**C**–**F**) The association between IPS and the risk model, the IPS, IPS-PD1, IPS-CTLA4, and IPS-PD1/CTLA4 scores were significantly increased in the low-risk group. Kaplan-Meier curve was used to analyze the survival of the high and low TMB load (**G**). The results showed that the survival of the two cohorts with high and low TMB load was significantly different. (**H**, **I**) Wilcoxon test was used to analyze the difference of PD1 and CTLA4 expression between high- and low-risk groups.

Using somatic mutation data available from the TCGA database, the relationship between mutation characteristics and this model was evaluated in BC patients. The frequently mutated genes in the high- and low-risk groups were presented in Figure 7A, 7B. It was intriguing that tumor mutation burden (TMB) was significantly higher in low-risk groups and associated with longer OS (Figure 7G).

### The immune-related risk signature and response to immune checkpoints-inhibitors (ICIs)

At last, we further explored the relationship between IPSips and this prognostic model. The IPS, IPS-PD1, IPS-CTLA4 and IPS-PD1/CTLA4 scores were designed to evaluate the feasibility of ICIs applying for BC patients. This result showed that the IPS was significantly increased in the low-risk group compared with the high-risk group (Figure 7C–7F). Moreover, the expression of PD1 and CTLA4 was higher in the low-risk group (Figure 7H, 7I). These results collectively suggested that IPS levels were higher in the low-risk group, and that these patients exhibited more immunophenotypes and were peculiarly prone to benefit from immune checkpoint therapy.

### The validation of immune-related SDC1 expression in BC samples

**Figure 8:**
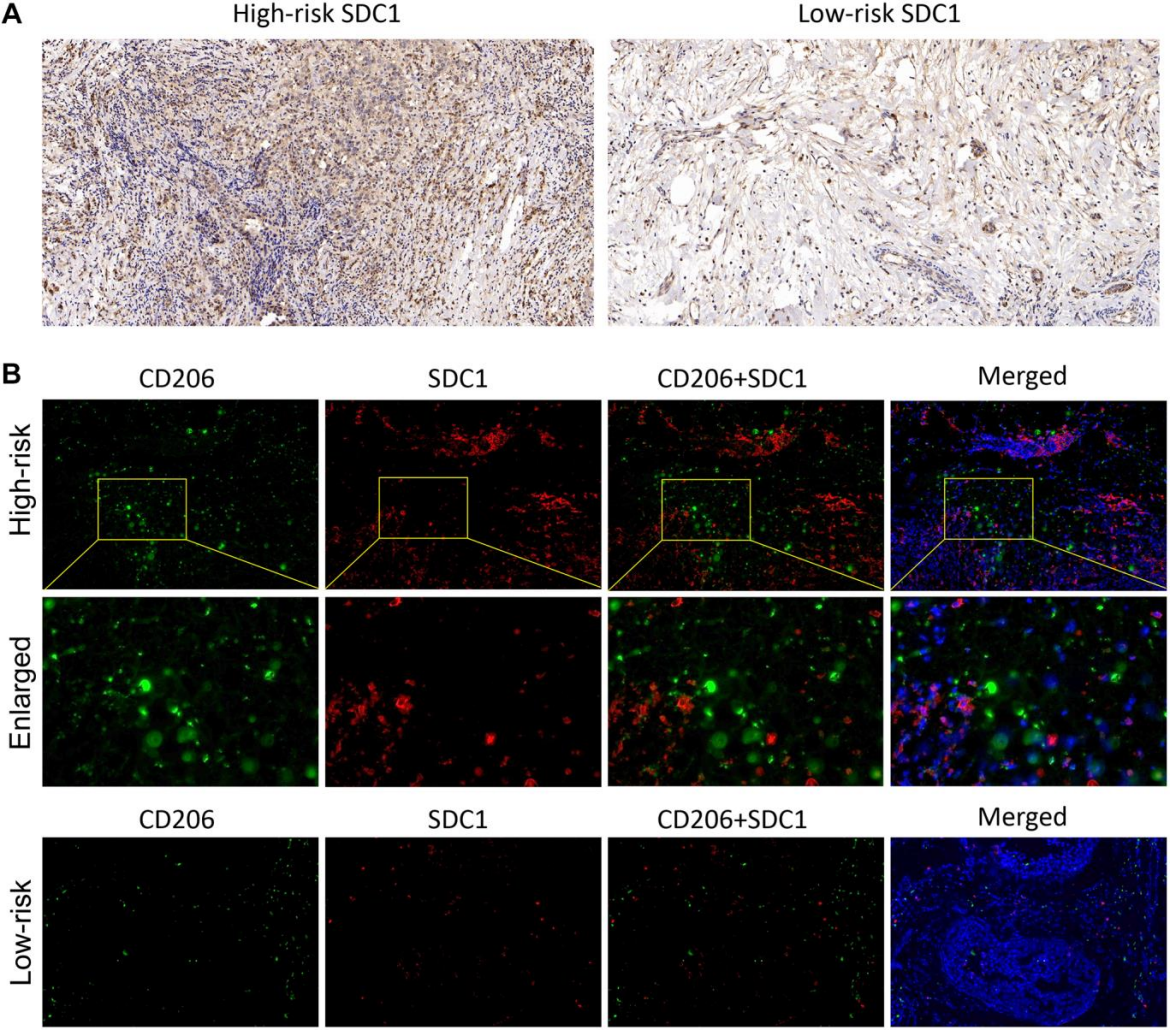
**The SDC1 expression features in BC samples.** (**A**) The IHC assay showed the SDC1 expression level in the high-risk and low-risk patients. (**B**) The IF assay verified the SDC1 and CD206 expression levels, as well as their co-expression in the high-risk and low-risk patients. CD206, green; SDC1, red; DAPI, nucleus.

In the end, the IHC and IF were performed to verify the SDC1 expression characteristics, which was one of the 6 IRGs in our risk model. The IHC results showed that SDC expressions were significantly higher in BC samples of high-risk patients, compared with those of low-risk patients (Figure 8A). Additionally, IF results showed the SDC1, as well as M2 macrophage biomarker CD206, were both of higher abundance in BC samples of high-risk patients, suggesting that SDC1 was indeed a tumor-promoting factor as previously reported and was positively linked to the expression of M2 macrophages (Figure 8B). The histological verification of BC samples successfully confirmed that the expression feature of IRGs in our model was in line with expectations.

## DISCUSSION

IRGs are a very critical category of genes that are not only involved in immune fine-tuning and tumor malignant progression, but also are very closely related to the prognosis of cancer patients. Therefore, the construction of IRGs-related prognostic prediction models for BC is of great scientific value and has the potential to be a useful supplement to conventional diagnosis and treatment. In this study, we successfully filtrated the differentially expressed mRNA in BC patients and accordingly screened 6 IRGs, including PSME2, ULBP2, IGHE, SCG2, SDC1, and SSTR1, to establish the prognostic model of BC patients. Then, we found the connection of the IRGs with clinicopathological characteristics, and different states of functional enrichment. More importantly, this IRG-based model had classical features in tumor-infiltrating immune cells and response to ICIs.

The existing tumor risk prediction models are still hot. The objects of interest used in different studies are varying, including IRGs, m6A-associated genes, death modality-associated genes, and others [23]. These characteristic models generated based on bioinformatics provide positive and meaningful strategies for clinical evaluation. Here, we focused on the model construction approach with IRGs as the main starting point, immune correlation, prognostic evaluation efficacy, and so on. For example, Chen et al. established a risk signature based on 8 differentially expressed IRGs for predicting the prognosis in squamous-cell lung cancer (SQLC) patients [24]. The risk score calculated from this model was able to accurately predict the prognosis and immune status of patients with this tumor. Yang et al. constructed a 11 IRG-constituting risk model in cervical cancer (CC) [25]. Their study highlighted that this risk model was an independent predictor of OS and progression-free interval, and the high-risk group classified therein was associated with lower numbers of CD8 T cells and resting mast cells. In addition, IPS analysis showed that the lower risk group with higher IPS indicated an immunogenic phenotype that was more prone to respond to ICI. In BC, Zhu et al. constructed a prognostic model using 12 IRGs to categorize BC patients into high-risk and low-risk groups [26]. Moreover, risk scores were adversely correlated with infiltration of B cells, CD4+ T cells, CD8+ T cells, neutrophils, and dendritic cells. In our study, the related results gave a consistent performance emphasizing that our prognostic model performs well in prediction. In the clinical direction, the constructed prognosis model also displayed the potential to predict the difference of prognosis between high- and low-risk groups, in age (≤ 65/> 65), clinical-stage (I and II/III and IV), T stage (T1, T2/T3, and T4), distant metastasis and lymph node metastasis (N0/NI and NII and NIII). These results indicated that our model was effective in predicting the BC prognosis under different clinicopathological conditions. Next, PCA analysis presented that our prognostic model based on immune gene expression and immune cell infiltration had a unique role in judging the prognosis of patients and rapidly adjusting the treatment plan. The degree of immune cell infiltration was negatively correlated with the prognosis risk score, indicating that low-risk patients were with more active immune state and better immune defense ability than high-risk patients. T and B cells play an important role in immune surveillance and tumor clearance. Combined with previous studies, it was speculated that the tumors with immune rejection phenotype were characterized by the presence of a large number of immune cells, which stayed in the matrix around the tumor cell nest and do not penetrate its parenchyma. The stromal activation in the high-risk group inhibited the anti-tumor effect of immune cells in BC.

SDC1 is essentially a heparan sulfate proteoglycan and a pivotal cellular adhesion protein that sustains the cell morphology and interaction with the surrounding microenvironment [27]. Moreover, SDC1 is engaged in malignant biological behaviors, including oncogenesis, invasion, metastasis, and angiogenesis a broad range of tumors, therefore being closely associated with tumor prognosis and therapy response. SDC1 also has a pro-metastatic role in the mouse model of breast cancer brain metastasis (BCBM), by which SDC1 regulates cytokines of the BBB and tames BC cells across the BBB [28]. For instance, by utilizing next-generation sequencing (NGS), Yeh et al. showed that SDC1 expression was negative with OS, suggesting that SDC1 might serve as a valid independent prognostic biomarker for breast ductal carcinoma [29]. A meta-analysis by Qiao et al. indicated that the overexpression of SDC1 protein in tumors was linked to a worse prognosis, including DFS and OS, and an aggressive phenotype is associated with negative ER expression and positive HER2 expression [30]. Here, our study confirmed coincident results that SDC1 possessed a high expression abundance in the high-risk patients, but not in the low-risk cohorts. Nevertheless, Qian’s study further pointed out the association between SDC1 and different BC subtypes of prognosis, which is lacking in our study, which is a superior thing to what we did in our study.

However, there are still many unresolved issues in terms of our study. Firstly, there are still ongoing reports of modeling studies related to IRGs that have shown good predictive performance. It is worth noting, however, that the number and genes of IRGs used in different studies are inconsistent and thus have some influence on the efficacy of the final models. Then, how to build the optimal number and gene of correlation models is a question worthy of in-depth consideration. Secondly, at present, our study is still mainly based on the existing database resources, which still need a large amount of external, clinical samples, and real-world data for further assessment. The credible external validation of other databases will be more convincing. Thirdly, although we have evaluated the SDC1 as the example to partially validate the efficacy of our IRGs model, the biological functions of the other 5 IRGs are also of necessity in regard to the model. It is also imperative to follow up with an in-depth and subsequent study on the relevant mechanisms. Therefore, there still has a long way in driving the clinically predictive value associated with this model.

## CONCLUSION

In conclusion, our study developed a novel risk score based on IRGs and its relationship with immune microenvironment, thus providing a predictive tool with considerable efficiency in clinical practice. Importantly, the correlations between IRGs and tumor immunity in BC warrant further investigation.

## Supplementary Materials

Supplementary Figures

Supplementary Tables
