# Supplementary material for: Development of a prognostic signature based on immune-related genes and the correlation with immune microenvironment in breast cancer
Source: Aging (Albany NY). 2022 Jul 5;14(13):5427–48. doi: 10.18632/aging.204158 (PMC9320535; doi:10.18632/aging.204158)
Supplement: Supplementary Figures [file aging-14-204158-s001.pdf]

SUPPLEMENTARY FIGURES

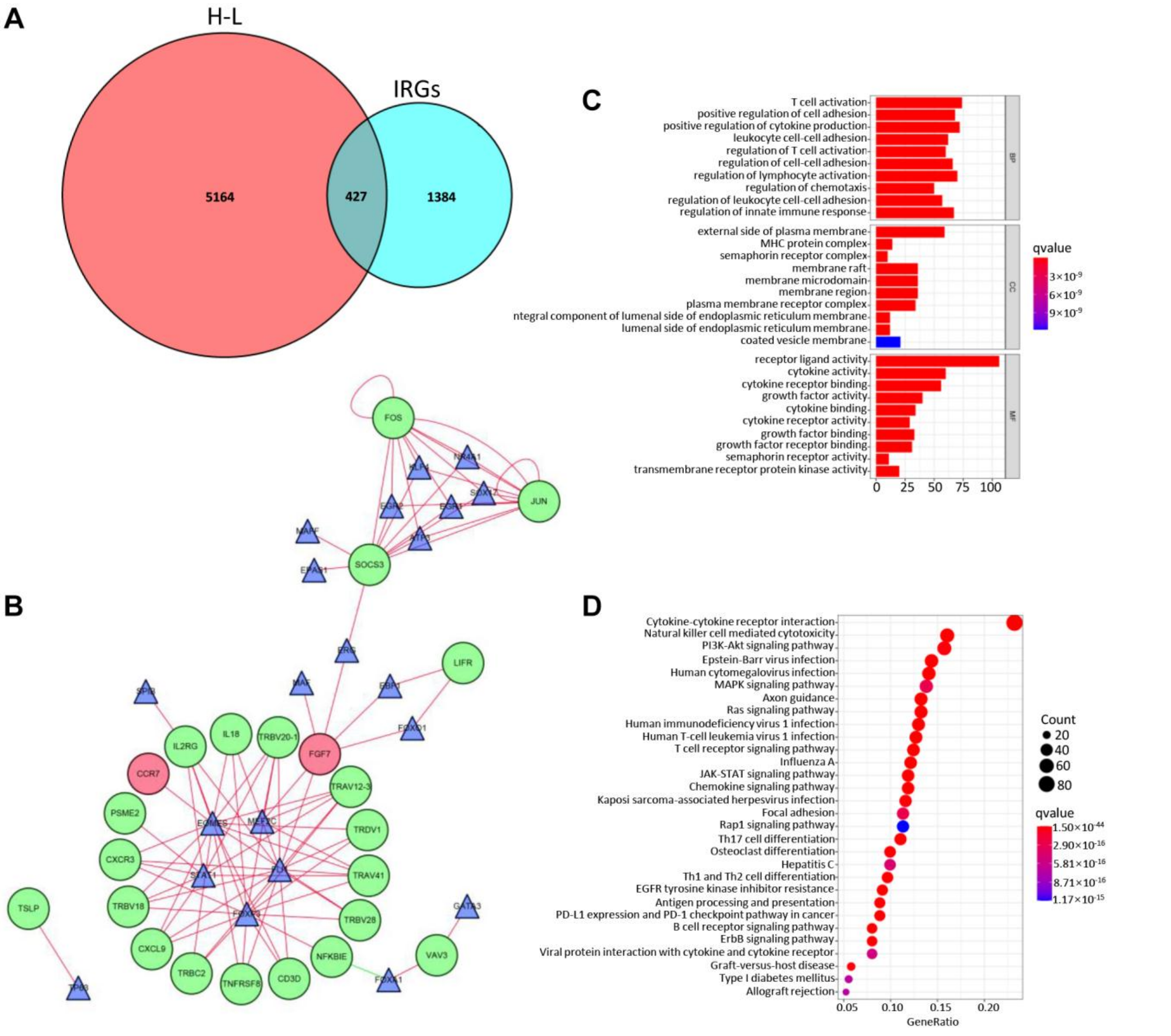

Supplementary Figure 1. The 427 immune-related differential genes in high- and low-risk groups were shown in Venn diagram (**A**). The regulatory network of transcription factors (TFs) and (IRGs). Regulatory networks based on survival-related TFs and IRGs in breast cancer. Pink circles indicated high-risk genes, green circles indicate low-risk genes, and purple triangles indicated transcription factors in the regulatory network (**B**). GO functional annotation (**C**) and KEGG (**D**) enrichment analysis were performed for the IRGs.

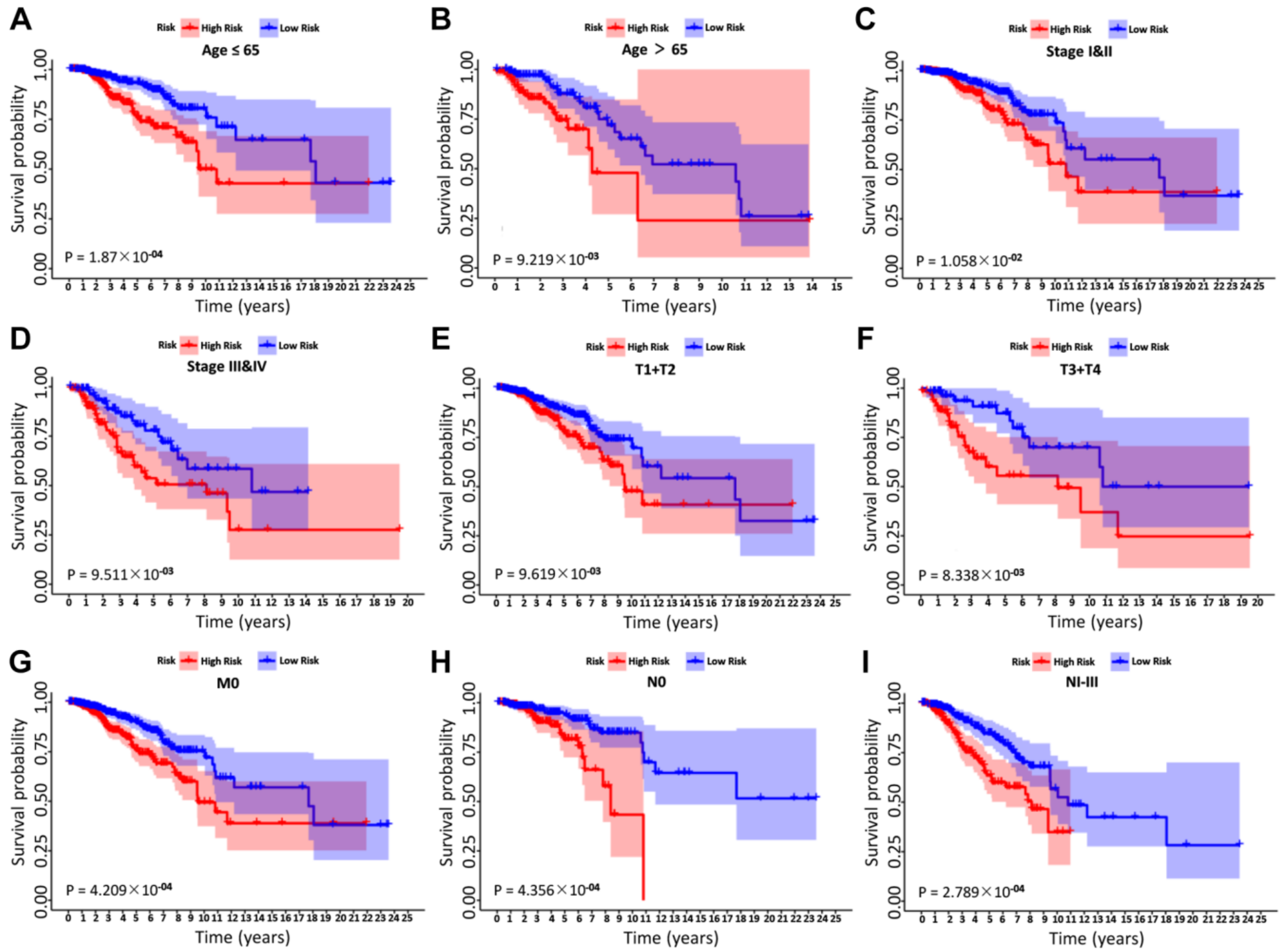

Supplementary Figure 2. The OS differences under different classification between the high-risk group and the low-risk group. Age (**A**, **B**), stage (**C**, **D**), T stage (**E**, **F**), distant metastasis (**G**) and lymph node metastasis (**H**, **I**).

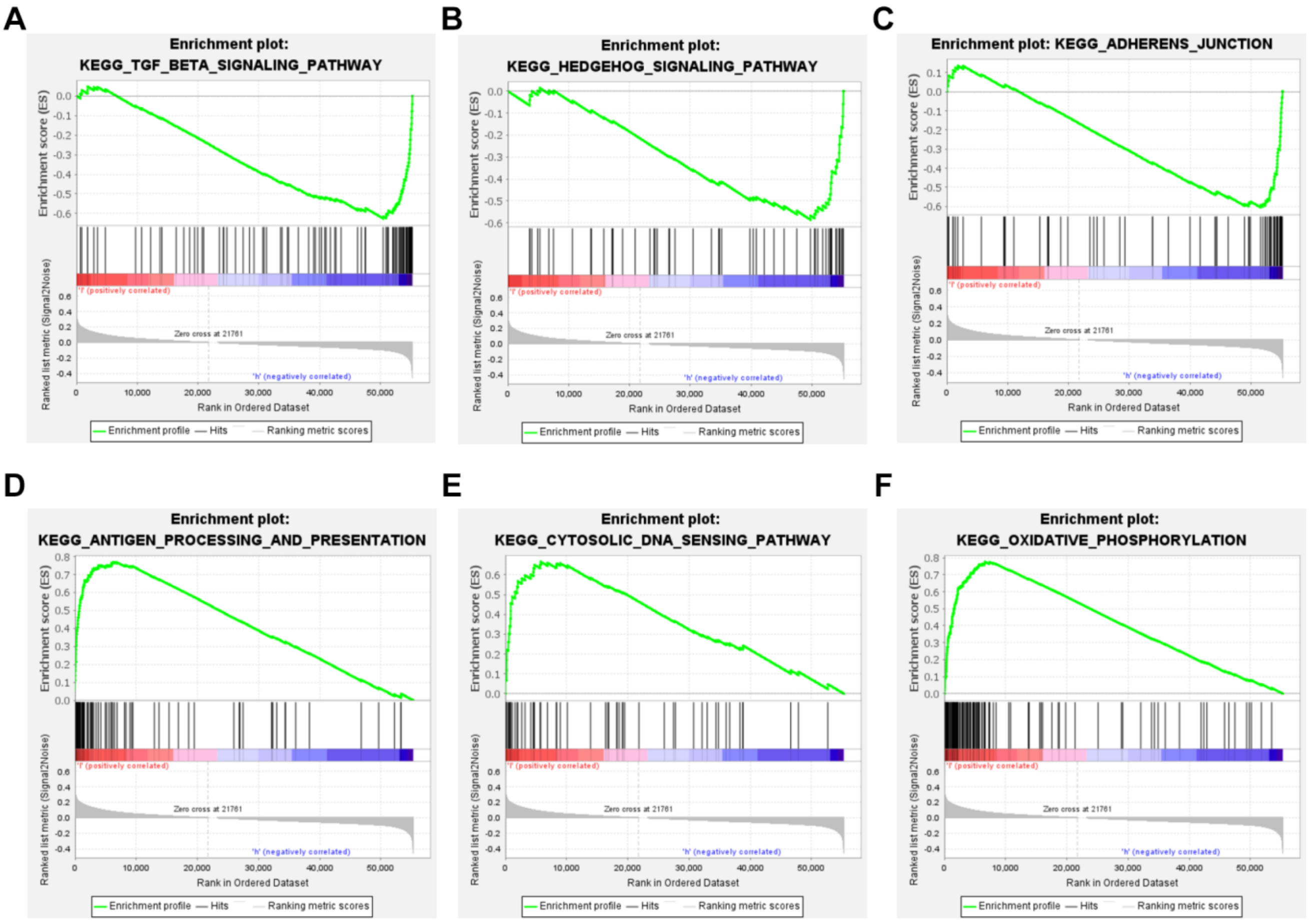

Supplementary Figure 3. KEGG pathway analysis from gene set enrichment analysis (GSEA). GSEA results showing (**A**), Hedgehog signaling pathway (**B**), Adherens junction enriched in the low-risk group (**C**), while Antigen processing and presentation (**D**), Cytosolic DNA sensing pathway (**E**), Oxidative phosphorylation were enriched in the high-risk group (**F**).
