# Supplementary material for: Development of a prognostic signature based on immune-related genes and the correlation with immune microenvironment in breast cancer
Source: Aging (Albany NY). 2022 Jul 5;14(13):5427–48. doi: 10.18632/aging.204158 (PMC9320535; doi:10.18632/aging.204158)
Supplement: Supplementary Tables [file aging-14-204158-s002.pdf]

SUPPLEMENTARY TABLES

Supplementary Table 1. General characteristics of breast cancer survival-related immune genes.

ID	HR	HR.95L	HR.95H	*P*-value
PSME2	0.98511606	0.97654026	0.99376718	0.00077519
ULBP2	1.12414412	1.05361278	1.199397	0.00040065
CXCL9	0.996502	0.9935673	0.99944536	0.01987772
CXCL13	0.99649413	0.99305749	0.99994265	0.04631633
S100A11	1.00025859	1.00002757	1.00048967	0.02824391
MMP9	1.00028343	1.00009984	1.00046705	0.00247757
PLAU	1.00520427	1.00132679	1.00909677	0.00847936
PLTP	1.00445817	1.00005618	1.00887953	0.04714064
SOCS3	0.9913671	0.98328682	0.99951378	0.03785364
JUN	0.99648985	0.99306484	0.99992667	0.04531569
IL18	0.93510883	0.88889297	0.98372758	0.00947637
TNFSF4	1.12112275	1.02672558	1.2241988	0.01084408
CCR7	1.01305579	1.00127558	1.02497459	0.02973711
CCL24	1.09096389	1.03150135	1.15385424	0.00233001
VAV3	0.99162762	0.98397554	0.99933921	0.03340306
FOS	0.99827108	0.99658311	0.99996191	0.04506121
NFKBIE	0.9613734	0.92951814	0.99432036	0.02194824
IGHE	1.05974172	1.0318211	1.08841787	2.05E-05
CXCR3	0.94015595	0.88939062	0.99381892	0.02934
ADM	1.01763819	1.00329978	1.03218151	0.01573582
FGF7	1.08206807	1.00944347	1.15991766	0.02607287
SCG2	1.00412622	1.00201544	1.00624145	0.00012544
TSLP	0.1923959	0.05487823	0.67451488	0.01001861
ADRB1	0.80855291	0.66812286	0.97849939	0.0290163
FLT3	0.88368991	0.80875706	0.96556542	0.00623679
IL2RG	0.98406789	0.97071868	0.99760068	0.02118435
LIFR	0.93736412	0.87897813	0.99962839	0.04869056
NPR3	1.03653553	1.00729834	1.06662134	0.01396767
SDC1	1.00256793	1.00105283	1.00408533	0.00088839
SSTR1	1.04209544	1.02001597	1.06465285	0.0001608
TNFRSF8	0.51228195	0.31193119	0.84131632	0.00822689
CD3D	0.97714889	0.95766	0.9970344	0.02451843
TRAV12-3	0.80263302	0.66324643	0.97131282	0.02388121
TRAV41	0.70015573	0.50332291	0.9739633	0.03429223

**TRBC2**	0.98298393	0.96927987	0.99688174	0.01657655
**TRBV18**	0.81249942	0.66168806	0.99768358	0.04746785
**TRBV20-1**	0.94364961	0.89055263	0.99991236	0.04965428
**TRBV28**	0.96882537	0.94321751	0.99512847	0.02048896
**TRDV1**	0.66519769	0.49695342	0.8904013	0.00613947

Supplementary Table 2. Univariate Cox proportional hazards regression analysis of IRGs.

ID	HR	HR.95L	HR.95H	*p*-value
**PSME2**	0.985116	0.97654	0.993767	0.000775
**ULBP2**	1.124144	1.053613	1.199397	0.000401
**CXCL9**	0.996502	0.993567	0.999445	0.019878
**CXCL13**	0.996494	0.993057	0.999943	0.046316
**S100A11**	1.000259	1.000028	1.00049	0.028244
**MMP9**	1.000283	1.0001	1.000467	0.002478
**PLAU**	1.005204	1.001327	1.009097	0.008479
**PLTP**	1.004458	1.000056	1.00888	0.047141
**SOCS3**	0.991367	0.983287	0.999514	0.037854
**JUN**	0.99649	0.993065	0.999927	0.045316
**IL18**	0.935109	0.888893	0.983728	0.009476
**TNFSF4**	1.121123	1.026726	1.224199	0.010844
**CCR7**	1.013056	1.001276	1.024975	0.029737
**CCL24**	1.090964	1.031501	1.153854	0.00233
**VAV3**	0.991628	0.983976	0.999339	0.033403
**FOS**	0.998271	0.996583	0.999962	0.045061
**NFKBIE**	0.961373	0.929518	0.99432	0.021948
**IGHE**	1.059742	1.031821	1.088418	2.05E-05
**CXCR3**	0.940156	0.889391	0.993819	0.02934
**ADM**	1.017638	1.0033	1.032182	0.015736
**FGF7**	1.082068	1.009443	1.159918	0.026073
**SCG2**	1.004126	1.002015	1.006241	0.000125
**TSLP**	0.192396	0.054878	0.674515	0.010019
**ADRB1**	0.808553	0.668123	0.978499	0.029016
**FLT3**	0.88369	0.808757	0.965565	0.006237
**IL2RG**	0.984068	0.970719	0.997601	0.021184
**LIFR**	0.937364	0.878978	0.999628	0.048691
**NPR3**	1.036536	1.007298	1.066621	0.013968
**SDC1**	1.002568	1.001053	1.004085	0.000888

**SSTR1**	1.042095	1.020016	1.064653	0.000161
**TNFRSF8**	0.512282	0.311931	0.841316	0.008227
**CD3D**	0.977149	0.95766	0.997034	0.024518
**TRAV12-3**	0.802633	0.663246	0.971313	0.023881
**TRAV41**	0.700156	0.503323	0.973963	0.034292
**TRBC2**	0.982984	0.96928	0.996882	0.016577
**TRBV18**	0.812499	0.661688	0.997684	0.047468
**TRBV20-1**	0.94365	0.890553	0.999912	0.049654
**TRBV28**	0.968825	0.943218	0.995128	0.020489
**TRDV1**	0.665198	0.496953	0.890401	0.006139
